# Bridging Lab and Life: A Dual‐Person Paradigm for Social Interaction Research

**DOI:** 10.1111/psyp.70292

**Published:** 2026-04-08

**Authors:** Vanessa Nöring, Lea Krismann, Marcel Franz, Fabian Rottstädt, Ilona Croy

**Affiliations:** ^1^ Department of Clinical Psychology Friedrich Schiller University Jena Jena Thuringia Germany; ^2^ German Center for Mental Health Halle‐Jena‐Magdeburg Germany

**Keywords:** dyadic interaction, interpersonal synchrony, methodological validation

## Abstract

Interpersonal synchrony, the spontaneous coordination of actions, emotions, and physiological processes, plays a crucial role in social bonding. While existing research has significantly advanced our understanding of interpersonal synchrony, many studies rely on either artificial paradigms that may limit ecological validity or naturalistic approaches that can pose challenges for achieving sufficient statistical power. To address this, we developed and validated a semi‐naturalistic verbal social interaction paradigm, in which two individuals talk about a variety of standardized neutral and autobiographical emotional topics. Sixty‐nine dyads (*N* = 138) participated and reported valence, arousal, connectedness, and dominance after each trial. Results confirmed the paradigm's reliability and validity by showing that emotional conversations, especially when sharing one's own autobiographical event, elicited higher arousal, more valence, and increased heart rate than neutral ones, with relatively stable affective responses across trials. Participants also felt more connected during emotional conversations, particularly with closer friends. In conclusion, we are optimistic that our social interaction paradigm is a useful tool for studies of dual person neuroscience by balancing experimental control and ecological validity. Its flexible design allows for adaptation across research contexts and supports broad application. This validation establishes the paradigm's reliability, enabling future hypothesis testing regarding interpersonal synchrony.

## Impact Statement

This study introduces and validates a novel semi‐naturalistic paradigm for examining interpersonal synchrony during real‐time social interaction. By balancing experimental control with ecological validity, our approach addresses critical limitations of prior paradigms. Its flexible design and reliable affective responses make it a valuable tool for research on neural and autonomic synchrony, supporting broad applications in social, affective, and interpersonal neuroscience and psychology.

## 1 Introduction

Social interactions are crucial for fulfilling the fundamental human need for belonging through regular contact and stable relationships (Baumeister and Leary 1995). Despite the fundamental role of human relationships in all aspects of life, neuroscience has traditionally focused on highly individualized approaches, leaving the brain processes involved in social interaction less thoroughly explored. Only recently has a shift toward a second‐person approach brought the study of social interaction into the spotlight (De Felice et al. 2025; Wheatley et al. 2024).

Within this framework, interpersonal synchrony has emerged as a particularly promising field for understanding interpersonal dynamics. Interpersonal synchrony refers to the spontaneous coordination of actions, emotions, thoughts, and physiological processes between two or more individuals (Mayo and Gordon 2020). Importantly, interpersonal synchrony constitutes one form of interpersonal coordination and is conceptually distinct from deliberate or strategic coordination, in which interaction partners intentionally align their behavior to achieve shared goals. While intentional and spontaneous forms of coordination may co‐occur within the same interaction, interpersonal synchrony specifically captures rhythmic and temporally coupled dynamics that emerge without explicit planning or instruction. Interpersonal synchrony is frequently observed in human behavior and has been proposed as an evolutionary‐based mechanism that strengthens social bonds by encouraging prosocial behavior and group cohesion (Launay et al. 2016). Despite the growing interest in interpersonal synchrony, existing research lacks a naturalistic social interaction paradigm, and our aim is to propose and validate such a framework to advance the study of synchrony in real‐world interactions.

Early examples of influential paradigms to explore interpersonal synchrony often require participants to engage in synchronized movements or actions, such as the finger tapping task (Hove and Risen 2009; Oullier et al. 2008) or the rocking chair study (Richardson et al. 2007), where participants synchronize their movement with another person. While such tasks provide valuable insights into synchronization processes, they lack the complexity of real‐life social interactions and artificially induce synchronization, limiting our understanding of the conditions under which high levels of interpersonal synchrony naturally emerge and why.

More naturalistic scenarios, as reviewed by Kelsen et al. (2022), include tasks such as back‐to‐back or face‐to‐face monologues and dialogues (Jiang et al. 2012), leaderless group discussions (Jiang et al. 2015), cooperatively planning a “fun” day with a stranger or romantic partner (Kinreich et al. 2017), or team brainstorming on a real‐world issue (Lu et al. 2019). While these paradigms resemble naturalistic interpersonal interactions well, they are not structured in a way that allows for the necessary power and control over emotional variation, both of which are essential for a comprehensive understanding of how social contexts modulate physiological states.

Emotional experiences play a fundamental role in social relationships, as emotions have a communicative function that facilitates interpersonal connection. For example, facial expressions of emotion provide information about socially relevant traits such as another person's cooperativeness and trustworthiness, or about their current goals and intentions (Van Kleef and Côté 2022). Therefore, many studies investigate the relationship between emotion and interpersonal synchrony, but they often do so by external emotion induction rather than studying emotion arising from social interaction itself. For instance, participants may watch emotional video clips (Fujiwara and Daibo 2018) or receive feedback on their performance (Smykovskyi et al. 2022). Although such approaches provide first insights, they do not fully capture how emotions naturally emerge and lack the interactive, dynamic quality of real social encounters (Gilam and Hendler 2016). This prompts a move toward paradigms that integrate natural emotional sharing within a conversational context.

Social sharing of emotion is a form of self‐disclosure focused on conveying emotional experiences. This process has been found to rekindle associated feelings within the sharer while also eliciting these emotions in the receiver, creating a reciprocal dynamic of affective alignment. Co‐experiencing emotions fosters cohesion and strengthens social bonds (Rimé 2009; Rimé et al. 2020), suggesting that emotional synchrony may serve as both a consequence and a catalyst of broader interpersonal synchrony mechanisms. Beyond the interpersonal level, recalling emotional episodes—particularly when expressed verbally—has also been associated with increases in heart rate, indicating heightened arousal in the speaker (Brugnera et al. 2018). More broadly, research shows that experiencing negative and positive emotions tends to elicit heart rate increases (Fernández et al. 2012; Shapiro et al. 2001; Simon et al. 2021). Together, these findings suggest that conversational sharing of negative experiences evokes elevated physiological responses, especially in the disclosing individual.

Beyond interpersonal synchrony research, studies have already incorporated emotion elicitation techniques, such as autobiographical recall, alongside social sharing of emotions within naturalistic interactions. For instance, Blanke et al. (2015) had dyads engage in a 12‐min conversation, during which each partner shared two personal experiences, one involving a positive emotional event and one involving a negative emotional event. Each participant had 3 min to discuss their experience while the partner listened and responded naturally. This design allowed the elicited emotions to unfold within a socially interactive setting, bridging the gap between controlled emotion induction and real‐world emotional dynamics.

Designing naturalistic paradigms involves a trade‐off between experimental control and ecological validity. Highly structured tasks offer experimental control but may lack the richness of real‐life interactions. On the other hand, more naturalistic paradigms (e.g., Blanke et al. 2015; Jiang et al. 2012, 2015; Kinreich et al. 2017), allow participants to see, hear, and respond to one another freely, capturing authentic emotional dynamics but also introducing challenges in data interpretation. Loosening the structure of experiments enhances generalizability but increases the complexity of analysis and the potential for artifacts and confounds, especially considering the small number of repetitions often found in these designs. Navigating this balance is crucial for advancing interpersonal synchrony research.

In response to this, our study introduces an ecological semi‐naturalistic verbal social interaction paradigm centered on structured autobiographical conversation where interaction partners engage in multiple short exchanges about emotionally negative personal events and standardized neutral topics. Our goal is to validate the paradigm's ability to reliably instantiate the psychological and physiological prerequisites of social interaction which serve as the necessary building blocks for higher‐order phenomena like interpersonal synchrony. By focusing on these foundational states rather than testing for the presence of synchrony itself, we provide a transparent, high‐quality baseline for future investigations into interpersonal coupling. Establishing this foundational construct validity ensures that the paradigm provides a stable and standardized platform for the field.

Hence, we tested the plausibility, reliability and validity of the paradigm, and expected that (1) conversations about emotionally negative personal events elicit more negative valence and higher arousal, as well as higher heart rate compared to neutral topics, and that (2) heart rate is positively associated with self‐reported arousal and extremity of valence. We further expected that (3) sharing one's own personal event will elicit a stronger emotional response and higher heart rate than listening to the other's event, (4) affective responses and heart rate patterns are stable within individuals across multiple trials, and (5) topic presenters are more dominant than their counterparts. Moreover, we expected that (6) experienced connectedness is higher during negative interactions, and higher in well‐befriended participants.

## 2 Methods

### 2.1 Participants

Participants were invited together as pairs of friends and a total of 69 dyads, or 138 individual participants, took part (107 female, 29 male, 2 other), aged between 18 and 30 years (mean 22.3y ± 2.3). Participants were required to have sufficient knowledge of German. Current or chronic neurological or psychiatric disorder and age below 18 or above 65 years served as exclusion criteria. Participants were recruited via university‐based student pools. The majority of participants (*n* = 134) indicated being enrolled in university, 78.4% of these being psychology students. On average, participant dyads had been friends for 2.6 years (±4.5) at the time of the study, with a range between 1 month and 27.3 years of friendship. There was no restriction with respect to gender composition of the dyads. Of the 52 same‐gender dyads, seven were male. The remaining 17 dyads were mixed‐gender.

### 2.2 Sample Size

To determine the required sample size, an a priori power analysis was conducted using G*Power (v3.1; Faul et al. 2007). We specified an F‐test for a repeated measures interaction within a 2 × 2 within‐subjects design (Condition and Topic Presenter). This configuration served as the closest approximation to our multilevel modeling approach, as both models evaluate variance within the same individual across conditions. The ‘correlation among repeated measures’ parameter (0.8) was utilized as a proxy for the random intercept, accounting for the inherent nesting of data points within participants. With a small effect size (*f* = 0.1), *α* = 0.05, and a power of 0.80, the analysis indicated a required minimum sample size of *N* = 56 individuals.

While this baseline estimate suggested a smaller cohort, we opted for a target of 50 dyads (*N* = 100 individuals) for two reasons. First, the increased sample size was necessary to adequately account for the higher complexity of the nested data structure and to ensure stable estimation of the random intercepts. Second, a larger recruitment goal provided a buffer against the high attrition rates common in dyadic research. Ultimately, we over‐recruited a total of 69 dyads (*N* = 138 individuals), providing increased sensitivity for detecting behavioral effects and ensuring the study was sufficiently powered to detect even small interaction effects.

### 2.3 Study Procedure

Before coming to the lab, each participant was asked to provide five emotionally negative and two positive events from their own lives which they felt comfortable discussing with their interaction partner. We focused on negative events to keep the design simple and time‐efficient, and because our primary interest was in affective synchrony more generally rather than the specific direction of emotion. The positive topics were used both to help participants practice before the main experiment and to ensure the conversation ended on a pleasant note. They were further asked to rate the valence and arousal of these events using the Self‐Assessment Manikin (SAM; Bradley and Lang 1994).

In the lab, participants first completed a set of demographic questions, followed by an in‐house social inclusion questionnaire and the German translation of the Perth Alexithymia Scale (Kaemmerer et al. 2021). Afterward, they received instructions on the study procedure and participated in the social interaction paradigm. During the interaction, we recorded video footage of each participant and measured brain blood oxygenation (fNIRS), ECG, and respiration. In the present manuscript, we focus on autonomic data, especially on the ECG‐derived heart rate data. Respiration was excluded from the current analysis due to significant confounds introduced by speech production. As noted in respiratory research (e.g., Fuchs and Rochet‐Capellan 2021), the act of speaking requires a transition from autonomic ‘tidal’ breathing to ‘speech breathing’, where respiratory rhythm is primarily governed by linguistic structure and phonatory needs. Because parameters such as inhalation depth and rate are heavily adapted to anticipate sentence length and support turn‐taking, they no longer serve as reliable proxies for emotional arousal in paradigms involving speech. The fNIRS and video recordings were not included in this validation paper as they are intended for a separate, more detailed analysis. Specifically, these data will be used in a future publication focusing on interpersonal synchrony and the neural and behavioral coordination between participants.

### 2.4 Social Interaction Paradigm

To develop a paradigm that balances ecological validity with experimental control, we designed a semi‐naturalistic social interaction task incorporating repeated conversational exchanges between friends. By structuring conversations around both standardized neutral topics and personally meaningful emotional experiences, this design aims to capture key features of real‐world social exchanges while ensuring consistency across trials. Multiple repetitions provide the necessary statistical power to assess dynamic changes in synchrony, while pre‐selecting personal events ensures that emotionally salient experiences are readily accessible. At the same time, we aimed to balance the need for sufficient repetitions to maintain statistical power with keeping the experiment's overall length manageable, preventing it from becoming excessively long. Our approach aims to allow for the spontaneous unfolding of emotion and interpersonal synchrony within a real conversational context.

**FIGURE 1:**
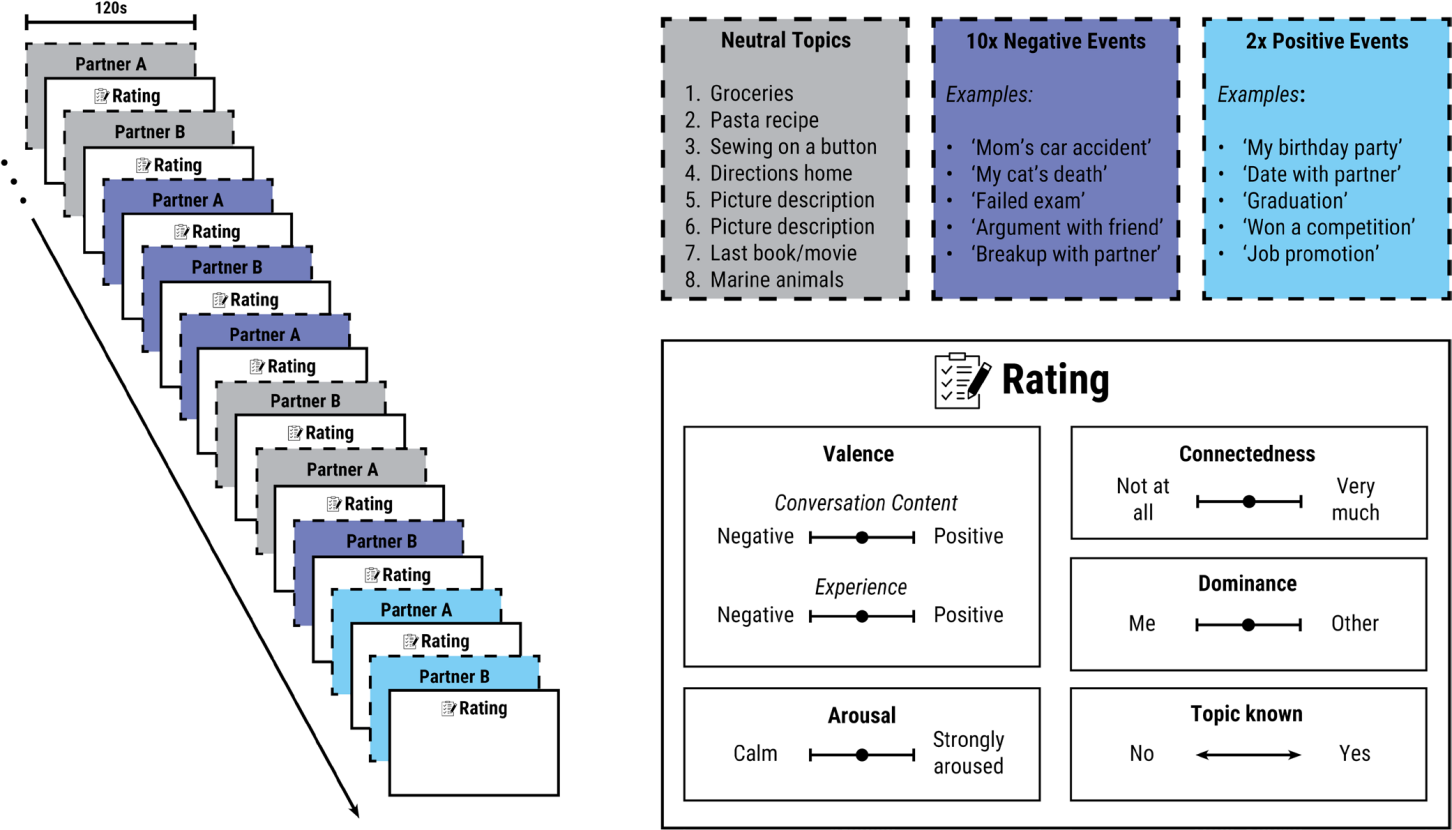
Social interaction paradigm. Schematic representation of the last ten out of twenty trials of the social interaction paradigm. Interaction partners discussed two standardized neutral topics and three autobiographical negative topics in an alternating sequence. This cycle was repeated three times, followed by two neutral topics and one negative topic, resulting in a total of eight neutral and ten negative trials. To conclude the conversation on a pleasant note, two positive events were discussed. The role of topic presenter was alternated between partners. After each trial, participants rated valence, arousal, connectedness, dominance, and whether they had already spoken about the topic with each other before participation.

Participants were instructed to discuss topics of different emotional valence with each other as naturally as possible within the experimental setting and to engage emotionally with the personal topics. These topics consisted of participant‐provided personal events and standardized neutral topics, which were presented in a fixed sequence across dyads (Table 1; Figure 1). Interaction partners took turns in presenting and discussing their respective topics. Using Presentation (Neurobehavioral Systems 2023), each topic and designated presenter were shown to the dyad on a screen for 12 s, after which a beep signaled the start and end of the conversation.

**TABLE 1:** Standardized neutral topics of the social interaction paradigm.

Trial	Speaker	Task (German)	Task (English)
1	A	Stelle dir vor, du planst jetzt deinen wöchentlichen Lebensmitteleinkauf. An was musst du alles denken? Was willst du alles einkaufen? Teile deine Gedanken mit deinem Gegenüber	Imagine you are planning your weekly grocery shopping now. What are you thinking of? What do you want to buy? Share your thoughts with the other person
2	B	Bitte erläutere deinem Gegenüber, wie du dein Lieblingspastagericht zubereiten würdest. Erläutere so präzise wie in einer Schritt‐für‐Schritt Kochanleitung	Please explain to the other person how you would prepare your favorite pasta dish. Explain as precisely as you would in step‐by‐step cooking instructions
6	B	Bitte erläutere deinem Gegenüber, wie man einen Knopf an ein Kleidungsstück annäht. Gehe davon aus, dass dein Gegenüber eine solche Tätigkeit noch nie zuvor gemacht hat und nicht weiß, was er dafür alles benötigt und wie man richtig näht	Please explain to the other person how to sew a button onto an item of clothing. Assume that the other person has never done this before and does not know what they need and how to sew correctly
7	A	Bitte erläutere deinem Gegenüber deinen Weg nach Hause nach Ende dieser Studie (z.B. Wie lange brauchst du zu dir nach Hause? Wo führt der Weg lang? Musst du dafür Verkehrsmittel nutzen? Gibt es gefährliche Kreuzungen? …)	Please explain your way home after this study to the other person (e.g., how long will it take you to get home? Where does the route take you? Do you have to use transportation? Are there any dangerous crossings? …)
11	A	Auf dem Tisch neben dir liegt ein umgedrehtes Bild, welches du dir nun anschauen darfst. Bitte beschreibe deinem Gegenüber sehr ausführlich und detailliert, was auf dem Bild zu sehen ist	There is a covered picture on the table next to you, which you may now look at. Please describe to your counterpart in great detail what can be seen in the picture
12	B	Auf dem Tisch neben dir liegt ein umgedrehtes Bild, welches du dir nun anschauen darfst. Bitte beschreibe deinem Gegenüber sehr ausführlich und detailliert, was auf dem Bild zu sehen ist	There is a covered picture on the table next to you, which you may now look at. Please describe to your counterpart in great detail what can be seen in the picture
16	B	Bitte erzähle deinem Gegenüber vom letzten Buch/Film, das/den du gelesen oder gesehen hast	Please tell the other person about the last book/movie you read or watched
17	A	Bitte nenne so viele Lebewesen wie möglich, die dir zur Kategorie “Meerestier” einfallen	Please name as many creatures as possible that you can think of in the category ‘marine animal’

Each trial had a fixed duration of 120 s. The trial duration was based on pilot data indicating that participants naturally spoke for at least 120 s when discussing emotional topics (see supplement). Additionally, in fNIRS hyperscanning, a frequency band of approximately 0.01–0.2 Hz is commonly used to capture neural synchrony (Chen et al. 2023; Cui et al. 2012; Pinti et al. 2019). To analyze coherence within this frequency range, wavelet transform coherence (Grinsted et al. 2004) requires a sufficient trial duration to provide meaningful data across relevant timescales. A 120‐s trial ensures that synchrony can be assessed within this band while allowing for naturalistic interaction.

Participants were allowed to naturally wrap up their conversation when the end signal occurred but were instructed not to continue speaking for an extended period beyond the signal. Whether conversations were cut off by the time limit varied substantially across dyads and topics. Trials were more likely to reach the time limit when topics were less familiar to the listening partner and required more contextual explanation, whereas familiar topics often led to shorter, more dialogic exchanges. Overall, inter‐dyad variability in narrative length and pacing was high, and participants frequently reported after the trial that they could have continued speaking.

The conversational format was intentionally naturalistic rather than strictly speaker‐listener based. Although one participant was designated as the primary topic holder, interaction partners were explicitly encouraged to ask follow‐up questions, provide comments, and interrupt as they normally would during everyday conversation. Deviations from the assigned topic were rare and typically brief. Interaction dynamics again depended on whether the topic was already familiar to both partners, with unfamiliar topics eliciting more extended monologic explanations and familiar topics leading to more interactive dialogue.

Topic valence followed a fixed sequence rather than being fully randomized. Specifically, two neutral topics were followed by three negative topics, and this cycle was repeated three times, followed by two neutral and one negative topic, resulting in a total of eight neutral and ten negative trials (Figure 1). Inspired by fMRT on–off design, this structure was chosen to avoid abrupt switches between emotional valences, to allow sufficient emotional immersion within negative blocks, and to reduce potential carryover effects from negative to neutral topics. While neutral and negative topics alternated at the block level, the sequence was not strictly alternating on a trial‐by‐trial basis. Furthermore, a fixed sequence provided a high degree of experimental standardization, which is essential for stabilizing the signal‐to‐noise ratio in interpersonal synchrony research where participant numbers are inherently constrained. After the final negative topic, two positive events were discussed. Those are not further analyzed but allow the conversation to be concluded on a pleasant note. Neutral and emotionally negative topics are alternated to help prevent emotional habituation over time, keeping affective engagement consistent.

After each trial, participants provided individual ratings of their experienced valence and arousal, the perceived valence of the conversation, their sense of connectedness to their partner, and their perceived conversational dominance. Additionally, they indicated whether they had previously discussed the topic with each other before the study. The ratings were conducted on tablets using the online survey tool LimeSurvey (LimeSurvey 2023).

For reasons of familiarization, the paradigm was preceded by two practice trials of personal positive events, followed by a short break to answer possible questions or solve any technical problems. The whole paradigm, including practice trials, took about 70 min per dyad. During the interaction, participants were placed on two chairs at a distance of approximately 90 cm, which were arranged at a diagonal angle to each other. The experimenter remained present in the room throughout the entire experiment to monitor the physiological measurements while sitting behind a room divider to minimize disturbances.

The paradigm was implemented with pairs of friends to facilitate spontaneous and emotionally meaningful self‐disclosure, which constitutes a core component of the paradigm. Prior work shows that familiarity increases emotional expressiveness (Clark and Finkel 2005), thereby enhancing ecological validity and ensuring that the intended psychological processes are reliably engaged. This choice is further supported by the fact that many emotionally relevant conversations in everyday life occur between familiar individuals rather than strangers (Rimé 2009; Rimé et al. 2020). Accordingly, the paradigm aims to model meaningful social interactions as they naturally occur, for which friends represent a common and ecologically valid dyad type, particularly in the context of autobiographical recall and emotional sharing.

### 2.5 Paradigm Development

The study design builds on insights from a pilot study (*n* = 14 dyads) that evaluated its feasibility, emotional content, and overall design (see supplement). Key findings from this pilot study informed several adjustments to the final design's paradigm structure. While the pilot demonstrated high ecological validity, a lower degree of standardization regarding question order and duration resulted in excessive variability in conversation timing across dyads, which complicated data analysis. Consequently, the final design implemented a fixed trial duration of 120 s and a predefined sequence to ensure higher standardization and more robust results. Secondly, the number of trials was increased to enhance statistical power while focusing exclusively on autobiographical negative events to make the design simpler and more time‐efficient. These events were pre‐selected by the participants at home to ensure immediate emotional access and salience. In contrast to the pilot, positive events were utilized only for a practice trial and at the end of the experiment to end on a pleasant note. To further facilitate deeper self‐disclosure, the final design recruited dyads of friends instead of strangers and included measures of friendship intimacy, while the standardized neutral conversation topics were adjusted and expanded (see Table 1). Regarding the dependent variables, valence and arousal (via Self‐Assessment‐Manikin) were retained but transitioned to participant self‐ratings, complemented by additional ratings for connectedness and dominance. The technical setup (comprised of Presentation software, video monitoring, fNIRS, ECG, and measures of respiration) proved successful in the pilot study and was adopted for the final design.

### 2.6 Measures

In line with the circumplex model of affect (Russell 1980), we measured emotional experience along two primary dimensions: valence (the degree of pleasantness vs. unpleasantness) and arousal (the level of physiological and psychological activation).

The dimensions arousal and valence were assessed using the pleasure and arousal subscales of the Self‐Assessment Manikin (SAM; Bradley and Lang 1994), which is a non‐verbal, visual tool for measuring a person's affective reaction to a stimulus. Arousal was rated on a scale from 0 to 8 and valence from −4 (negative) to 4 (positive). Connectedness was measured by asking ‘How strongly did you connect with your counterpart?’ on a scale from 0 = *not at all* to 8 = *very much* and dominance by asking ‘Who was more dominant during the conversation?’ on a scale from −4 = *Me* to 4 = *The Other*. Participants also dichotomously indicated whether or not they had already discussed a topic with their interaction partner before participating in the study.

### 2.7 ECG Measurement and Preprocessing

ECG signals were recorded at a sampling rate of 500 Hz using the RESP sensors of the NIRxWINGS module, integrated with the NIRSport2 setup. Two RESP paddles were positioned bilaterally on the participant's chest, just below the clavicles, following the manufacturer's guidelines. Additionally, a ground electrode was placed on the right hip.

R‐peaks were extracted from preprocessed ECG signals using NeuroKit2's default processing function (Makowski et al. 2021). To assess signal quality, we calculated the absolute differences between successive R–R intervals (ΔRR) within each 120‐s trial. Trials were excluded if more than 20% of ΔRR values exceeded a fixed threshold of 100 ms. Additionally, we removed trials with implausible mean heart rates (< 50 bpm or > 120 bpm) to ensure physiological validity. For each included trial (73.04% of the data), mean heart rate was computed using NeuroKit2, applied to the 120‐s epochs.

### 2.8 Statistical Analyses

All analyses were performed using *R Statistical Software* (R Core Team 2021). The cutoff value for Type I error was set to *α* = 0.05. Mixed models were implemented using the *lme4* package (Bates et al. 2015) and repeated measures correlations were conducted using the *rmcorr* package (Bakdash and Marusich 2016).

To account for the nested structure of the data, we initially sought to specify a maximal random‐effects structure including random intercepts and random slopes for the within‐person predictors. However, these maximal models yielded singular fits, suggesting that the data did not support the estimation of these additional variance components. Consequently, we adopted a more parsimonious structure, retaining random intercepts for individuals (Level 1) and, where justified by the variance, for dyads (Level 2).

#### 2.8.1 Arousal and Valence

We first tested whether negative compared to neutral trials and sharing one's own personal topic were experienced as more arousing and more negative. To account for the nested structure of our data, we employed a multilevel modeling approach and specified two models, one each for arousal and valence as dependent variables. The fixed effects in both models included condition, topic presenter and trial number (as a covariate). Condition consisted of two levels (negative, neutral) and was dummy‐coded with neutral as the reference category. Topic presenter also consisted of two levels (self, other) with other as the reference category. To determine if the effect of the emotional condition depended on who was presenting, we specified a two‐way interaction term of condition and topic presenter. In the valence model, we included repeated measures within individuals at Level 1 and dyads at Level 2 as random intercepts. Due to negligible variance in the random intercept of dyads, which caused a singular fit issue, the arousal model only included the random intercept for repeated measures within individuals.

To test whether valence and arousal can be reliably evoked across multiple trials, we calculated Cronbach's alpha across trials within individuals.

#### 2.8.2 Dominance

We also tested whether participants consistently agreed on who was more dominant in each trial. To do so, we conducted a repeated measures correlation, which assesses the association between two variables while accounting for within‐subject dependencies. Although this method specifically captures intra‐individual variance rather than modeling multiple sources of variance simultaneously (as a multilevel model with fixed and random effects would), it is conceptually similar to a null multilevel model with varying intercepts and a common slope for each individual (Bakdash and Marusich 2017). Given a sufficient degree of agreement, and considering the mirrored nature of the dominance score, one partner's dominance rating was reversed. This recoding allowed for the calculation of a dyad‐level measure of dominance by taking the mean of both participants' ratings. This dominance measure served as the dependent variable in a linear regression model where the predictor was a categorical variable indicating whose topic (own, other) was discussed. The category other served as the reference category. We chose to conduct a linear regression because the random intercept of dyads showed negligible variance, leading to a singular fit issue in a mixed‐effects model.

#### 2.8.3 Heart Rate

To test the hypotheses regarding participants' heart rate, we conducted a multilevel model with mean heart rate per trial as dependent variable. Condition, topic presenter, and trial number were included as fixed effects. Condition consisted of two levels (negative, neutral) and was dummy‐coded with neutral as the reference category. Topic presenter also consisted of two levels (self, other) with other as the reference category. To determine if the effect of the emotional condition depended on who was presenting, we specified a two‐way interaction term of condition and topic presenter. Due to negligible variance in the random intercept of dyads, which resulted in a singular fit, the final model only included a random intercept to account for repeated measures within individuals.

We further performed a repeated measures correlation for experienced arousal and heart rate, as well as for experienced valence and heart rate.

#### 2.8.4 Connectedness

In a subset of *n* = 111 participants (due to the later introduction of the friendship item in the study), we finally tested whether individuals feel more connected during negative trials and whether this relates to their report of intimate friendship. Prior to analysis, we examined the range of responses for connectedness. Participants' ratings fell between 3 and 6 on the original 0–6 scale, indicating that the lowest response options were not used. For clarity in interpretation, we rescaled the connectedness variable by subtracting the minimum observed value from all responses. As a result, the new scale ranged from 0 to 3, where 0 represented the lowest observed level of connectedness and 3 corresponded to the highest. This transformation ensured that the lowest observed level of connectedness was treated as the baseline in our analysis.

We then computed a multilevel model with connectedness as the dependent variable. The fixed effects were friendship intimacy and condition. Condition consisted of two levels (negative, neutral) with neutral as the reference category. We included an interaction term of condition and friendship intimacy to test whether the effect of the emotional condition on connectedness was moderated by the existing level of friendship intimacy. Repeated measures within individuals at Level 1 and dyads at Level 2 were included as random intercepts.

#### 2.8.5 Topic Content

Across all participants, 53.6% of the negative conversation topics had been discussed before taking part in the study. We furthermore explored the content of the personal events. Using a bottom‐up approach, twelve categories of conversation topics were created by screening the content of the personal negative events. The following requirements for the categorization were applied: (1) The categories should be mutually exclusive, (2) at least 5 conversation topics should be assignable to each category and (3) the categories should cover as many of the discussed topics as possible. The resulting categories were *occupation*, *health*, *living situation*, *death*, *self‐reflection*, *travels*, *politics*, *material goods*, *finances*, *accidents*, *driving lessons*, and *violence/harassment*. An additional category of *other* was used if the assignment to one of the chosen categories was not possible. The events were also divided into *social* and *not social* categories, describing whether another person was relevant for the event. For social topics, the rater also judged whether the event concerned a close relationship, an acquaintance or a stranger (based on Lang and Fingerman 2003; Marsden and Campbell 1984).

Two mixed models were calculated to test whether social versus non‐social topics related to experienced valence and arousal. The dummy‐coded variable topic content (two levels: social, non‐social) served as the dependent variable, with non‐social as the reference category. Regarding the random‐effects structure, we accounted for the nested nature of the data by specifying random intercepts for individuals (Level 1) nested within dyads (Level 2).

## 3 Results

**FIGURE 2:**
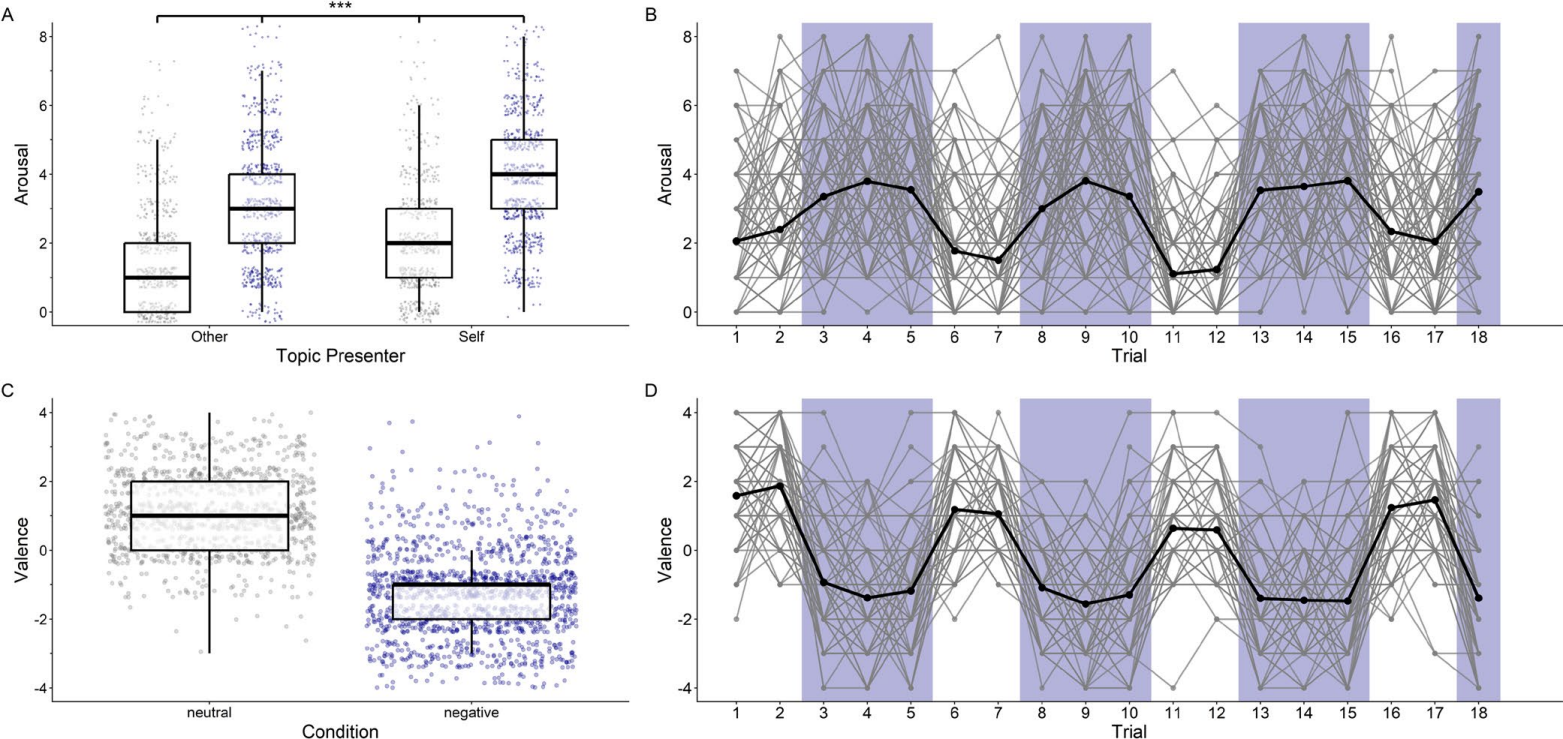
Arousal and valence across negative and neutral trials. ***p < 0.001. (A and C) depict boxplots and scatterplots of arousal by condition and topic presenter and valence by condition. Each scatter point represents an individual trial. The boxplot shows the median and interquartile range. All pairwise comparisons in Figure A were statistically significant. (B and D) display the trajectory of experienced arousal and valence across trials. The black line depicts the average. The ratings of each participant (*n* = 139) are visible in the background. On average, arousal was stably higher and valence was stably more negative during negative (marked blue [gray]) as compared to neutral trials. Estimated marginal means of arousal are presented in Table 2. Mean valence was −1.32 (±1.34) during negative and 1.20 (±1.20) during neutral trials.

Participants reported significantly higher arousal during negative conversations compared to neutral ones (*b* = 1.57, SE = 0.08, *t*(2342) = 18.75, *p* < 0.001), and when talking about their own experience compared to listening to their partner (*b* = 0.42, SE = 0.09, *t*(2342) = 4.75, *p* < 0.001). This effect was further qualified by a significant interaction between condition and topic presenter (*b* = 0.31, SE = 0.12, *t*(2342) = 2.59, *p* < 0.01), indicating that the increase in arousal during negative conversations was even more pronounced when participants were talking about their own negative experience rather than listening to their partner's. After Tukey adjustment, all pairwise contrasts were statistically significant (*p* < 0.001; Figure 2). See Table 2 for estimated marginal means. Trial number had no effect on reported arousal (*b* = −0.002, SE = 0.006, *p* = 0.71).

**TABLE 2:** Estimated marginal means of arousal and heart rate.

Condition	Role	Estimated arousal (Mean)	SE	df	95% CI
Neutral	Other	1.60	0.10	281.75	[1.40, 1.80]
	Self	2.02	0.10	281.75	[1.82, 2.21]
Negative	Other	3.17	0.10	240.25	[3.00, 3.36]
	Self	3.90	0.10	240.25	[3.71, 4.08]

Condition	Role	Estimated HR (Mean)	SE	df	95% CI
Neutral	Other	81.36	1.09	110.09	[79.20, 83.52]
	Self	84.34	1.10	112.34	[82.17, 86.51]
Negative	Other	81.31	1.09	109.00	[79.15, 83.46]
	Self	85.92	1.09	109.98	[83.76, 88.08]

Participants reported significantly more negative valence during negative conversations compared to neutral ones (*b* = −2.42, SE = 0.07, *t*(2342) = −36.31, *p* < 0.001; Figure 2). There was no significant main effect of topic presenter (*b* = −0.09, SE = 0.07, *t*(2342) = −1.34, *p* = 0.18). The interaction between condition and topic presenter was not significant (*b* = −0.16, SE = 0.09, *t*(2342) = −1.71, *p* = 0.087). Trial number was a significant negative predictor of reported valence, indicating a slight trend toward more negatively experienced valence over time (*b* = −0.03, SE = 0.005, *p* < 0.001).

**FIGURE 3:**
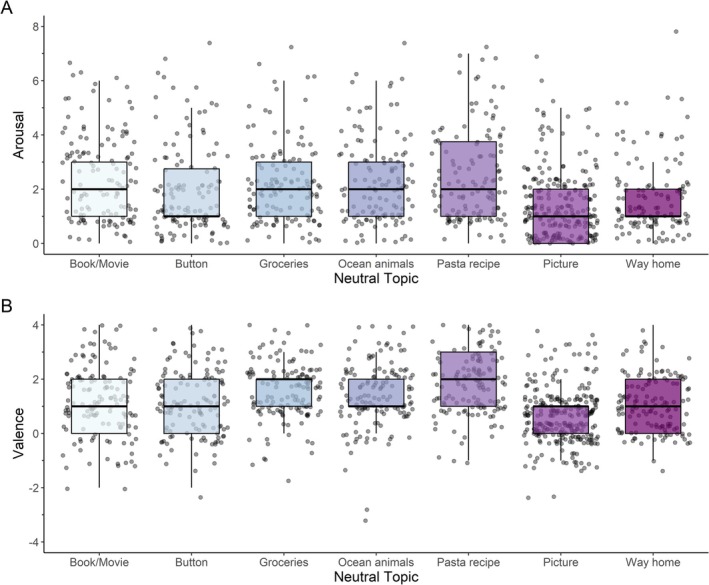
Arousal and valence by neutral conversation topic. Boxplot and scatterplot of arousal (A) and valence (B) ratings by neutral conversation topic. Each scatter point represents a rating by one individual. The boxplot shows the median and quartiles.

Over the course of the experiment, arousal ratings remained stable across trials, while a small but consistent time‐related shift in valence was observed. This temporal trend is also reflected in the lower (but still acceptable) internal consistency of valence ratings for neutral events (Cronbach's *α* = 0.71), compared to higher consistency for negative trials (*α* = 0.84 for both valence and arousal) and arousal in neutral trials (*α* = 0.82). Among neutral topics, the average reported valence ratings ranged from 0.62 to 1.86, indicating that while all topics were generally associated with a positive affective response, some—such as discussions about pasta recipes (1.86 ± 1.16) and grocery shopping (1.58 ± 1.08)—elicited higher positive valence than others, like describing a picture (0.62 ± 1.05) and one's way home (1.05 ± 0.97). Similarly, arousal levels varied across neutral topics, with the lowest arousal reported for the picture description (1.17 ± 1.32) and describing one's way home (1.50 ± 1.42), while the highest arousal was observed for the pasta recipe (2.40 ± 1.79) and talking about a recent book or movie (2.34 ± 1.71). For an overview of valence and arousal ratings by neutral topic, see Figure 3.

**FIGURE 4:**
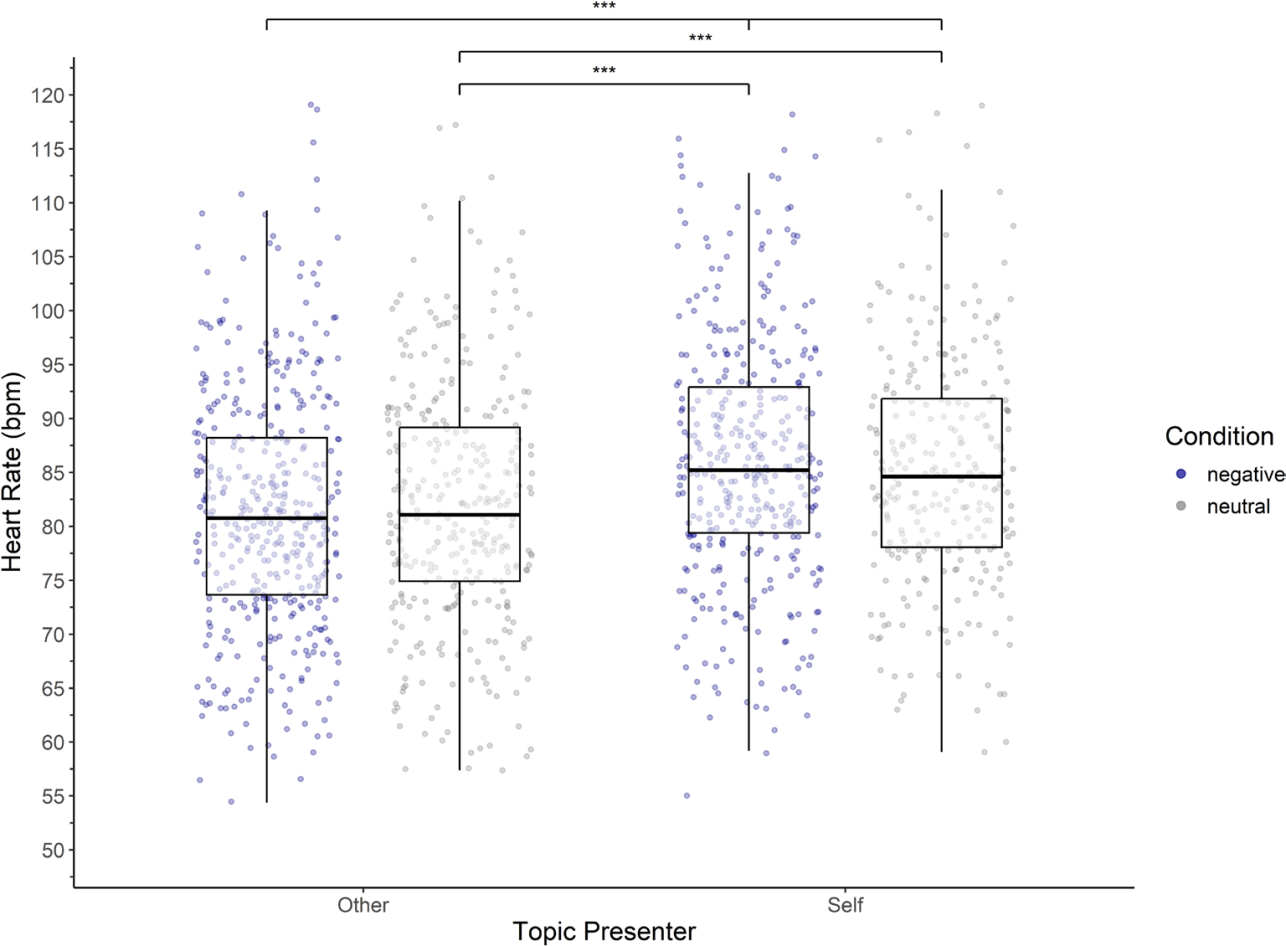
Heart rate by condition and topic presenter. ***p < 0.001. Boxplot and scatterplot of heart rate by condition and topic presenter. Each scatter point represents the average heart rate per trial and individual. The boxplot shows the median and quartiles. Pairwise comparisons were conducted using estimated marginal means. Except for other‐negative and other‐neutral, all contrasts were statistically significant.

Participants' heart rate was significantly elevated when they spoke about their own experiences compared to when they listened to their partner (*b* = 2.97, SE = 0.30, *t*(1313) = 10.03, *p* < 0.001). No main effect of condition was observed (*b* = −0.06, SE = 0.27, *t*(1314) = −0.22, *p* = 0.83). Crucially, there was a significant interaction between condition and speaker role (*b* = 1.64, SE = 0.39, *t*(1313) = 4.18, *p* < 0.001), indicating that the increase in heart rate during negative conversations was especially pronounced when participants themselves were speaking (Figure 4). Heart rate also showed a linear decline across trials (*b* = −0.34, SE = 0.02, *t*(1315) = −17.93, *p* < 0.001), suggesting habituation over time. Heart rate was significantly positively correlated with reported arousal (*r* = 0.23, *p* < 0.001) and with absolute values of reported valence (*r* = 0.10, *p* < 0.001). While statistically robust, both correlations reflect small effect sizes.

**FIGURE 5:**
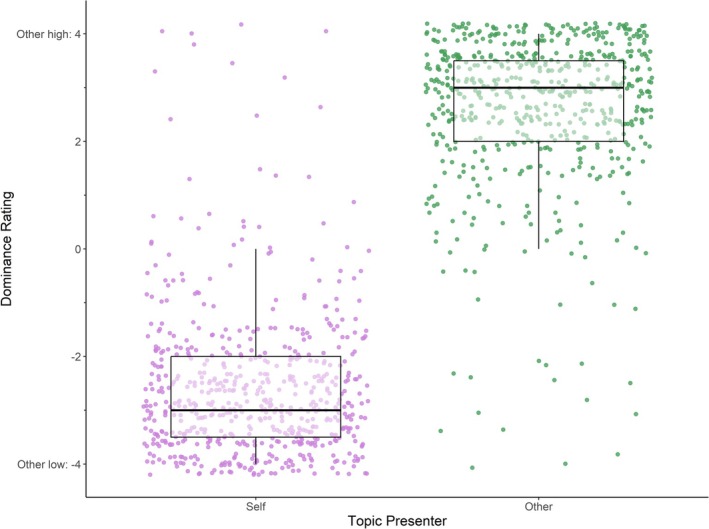
Dominance ratings by topic presenter. Boxplot and scatterplot of dominance ratings by topic presenter. Each scatter point represents a dyad and trial. Negative dominance values indicate low conversational dominance of the other, while positive values indicate high dominance of the other. On average, dominance ratings were −2.60 (±1.35) when presenting one's own topic and 2.65 (±1.37) when the other presented the topic.

We found a high level of agreement between interaction partners' ratings of dominance during trials (*r*(1172) = −0.92, *p* < 0.001), suggesting that individuals largely concurred on who was more conversationally dominant in each conversation. Participants reported being more dominant during trials of topics of their own as compared to topics presented by the other and vice versa (*b* = 5.25, SE = 0.08, *t*(1240) = 68.11, *p* < 0.001; Figure 5; see Table 3 for mean dominance scores).

**TABLE 3:** Mean and standard deviation of all relevant variables.

Variable	Condition: Negative		Condition: Neutral	
	*M*	SD	*M*	SD
Valence	−1.32	1.34	1.20	1.21
Arousal	3.53	1.84	1.81	1.62
Connectedness	4.95	1.86	3.88	1.97

	Presenter: Self		Presenter: Other	
	*M*	SD	*M*	SD
Dominance	−2.60	1.35	2.65	1.37

	*M*	SD
Friendship intimacy	4.68	0.97

*Note:* Negative dominance values indicate conversational dominance of the self while positive values indicate dominance by the other.

**FIGURE 6:**
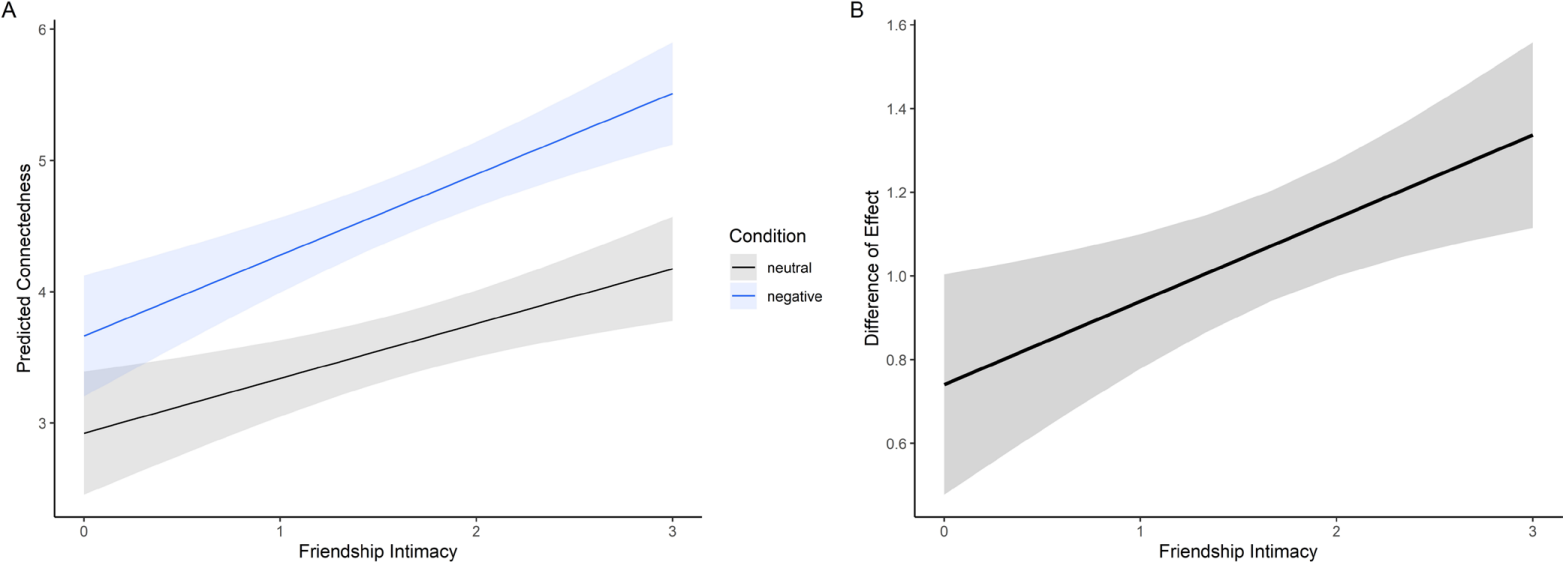
Model‐based predictions of connectedness by friendship intimacy and condition. (A) Average predicted connectedness as a function of friendship intimacy during negative versus neutral trials. The plot presents model‐based predictions, with lines and 95% confidence intervals (CIs) showing estimated connectedness levels based on the interaction between friendship intimacy and condition, derived from a linear mixed‐effects model. (B) Predicted difference in connectedness between the negative and neutral conditions across levels of friendship intimacy. The black line represents the estimated difference, with the shaded area indicating the 95% CI. For friendship intimacy, a value of 0 represents medium friendship intimacy, while a value of 3 indicates very close friendship intimacy.

People who considered their friendship to be particularly close also felt a stronger sense of connection while talking (*b* = 0.42, SE = 0.12, *t*(116) = 3.42, *p* < 0.001). Participants also reported feeling more connected to their counterpart during negative conversations compared to neutral conversations (*b* = 0.74, SE = 0.13, *t*(1868) = 5.52, *p* < 0.001). Additionally, the influence of friendship intimacy on feelings of connectedness was slightly higher during negative conversations (*b* = 0.20, SE = 0.07, *t*(1868) = 2.88, *p* < 0.01; Figure 6). Mean scores for connectedness and friendship intimacy are reported in Table 3.

**FIGURE 7:**
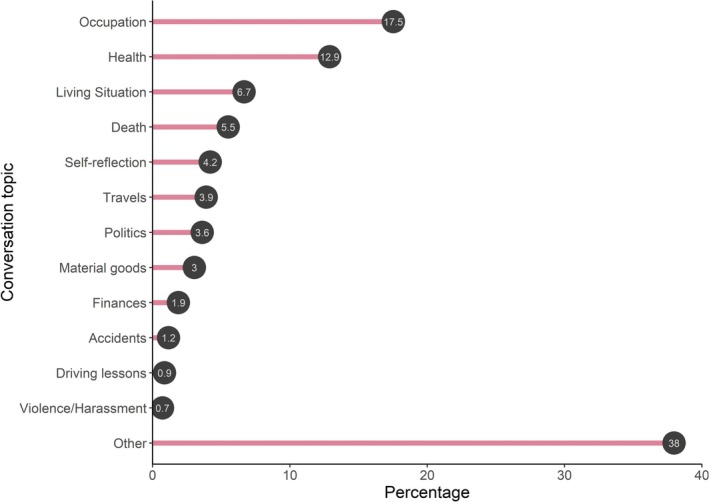
Negative conversation categories. Percentages of categories discussed during negative trials, based on the categorization of 700 conversation topics.

Most of the conversations which were designed to cover a negative personal topic concerned occupation in our mainly student sample (e.g., university or job‐related matters), followed by health (e.g., sickness, stress) or living situation (e.g., flat sharing, moving). Ranging between 0.7% and 1.2%, violence and harassment, driving lessons and accidents were the smallest specific categories discussed. However, most topics did not fall into any of those themes (Figure 7). The majority of the topics covered were social incidences (52.8%), a smaller portion non‐social incidences (38%), and 9.2% could not be classified as social or non‐social because we were unable to interpret the provided titles. Of the social topics, 74.7% concerned close relationships, 6.9% acquaintances and 3% strangers, with another 15.4% undefined. Talking about a social topic was significantly associated with a more negative experienced valence (*b* = −0.32, SE = 0.07, *t*(1161) = −4.74, *p* < 0.001) and a higher experience of arousal (*b* = 0.24, SE = 0.09, *t*(1169) = 2.62, *p* < 0.01).

## 4 Discussion

Interpersonal synchrony research faces a trade‐off between ecological validity and experimental control, with many existing paradigms either lacking the richness of real‐world social interactions or failing to provide sufficient statistical power for robust analyses. Our study aimed to develop and validate a semi‐naturalistic verbal social interaction paradigm that addresses this gap. By systematically integrating emotionally negative autobiographical recall with repeated conversational exchanges, we sought to balance ecological validity with experimental control, allowing for a more comprehensive investigation of synchrony in real‐world social interactions.

Discussing negative personal events was associated with greater arousal compared to talking about standardized neutral topics, an effect that was particularly pronounced when talking about one's own autobiographical event. Additionally, discussing a negative autobiographical event was also related to more negative affective responses. This is in line with evidence that negative autobiographical recall typically triggers negative emotions and increased arousal and that the social sharing of emotion elicits similar emotions in the receiver (Lench et al. 2011; Rimé 2009; Rimé et al. 2020). Although these findings generally suggest that the paradigm reliably evokes the expected levels of arousal and valence, the standardized neutral topics were experienced as slightly positive. This may be because participants interacted with friends or due to the nature of the tasks themselves. Alternatively, the use of positively valenced practice trials before the actual experiment might have had an influence on this as well. However, practice trials were temporally separated from the experimental trials, resulting in a substantial delay before the onset of the main task. While short‐lived affective priming effects cannot be fully excluded, the overall duration of the experiment (approximately 1 h) and the temporal stability of valence ratings make it unlikely that two brief positive practice trials induced sustained affective priming across all subsequent neutral trials. To rule out a possible influence, future studies building on this paradigm may opt for neutral practice trials.

While the contrast between negative and neutral conditions is still present, the mildly positive nature of the neutral topics can anyhow be problematic. The reason is that both negative and neutral conditions evoke valence, namely negative and positive valence. If one aims to study effects of valence—irrespective of its emotional tune—the current neutral condition is not a suitable baseline. To address this, the current standardized topics could be exchanged for more neutral alternatives, such as picture description tasks only, which were rated as particularly neutral in our study and low in arousal compared to other topics. Future studies may benefit from exclusively using such prompts or incorporating similar low‐arousal ones to ensure a clearer emotional contrast. Alternatively, if interested in comparing negative to positive valence, one might also consider using positive autobiographical events instead of neutral topics. In the present study, however, we opted against this for reasons of simplicity, time constraints (to avoid unnecessarily prolonging the experiment for participants), and a specific focus on examining affective synchrony in contrast to emotionally flat or neutral states—making the exact valence (positive or negative) less central.

While arousal ratings remained stable across trials—indicating no signs of fatigue or habituation—valence ratings became increasingly negative over time. This shift reflects a growing adaptation to the neutral trials, which were rated as slightly positive overall. As participants became more accustomed to the interaction setting or the nature of the neutral tasks, these may have lost some of their initial lightness or amusement and come to feel more emotionally flat. In line, internal consistency for valence ratings during neutral trials was somewhat lower. Since we did not include more than ten trials per condition, we cannot determine whether a higher number might have helped reduce the perceived positivity of the neutral trials. On the one hand, additional repetitions could have increased the stability of affect ratings and improved the contrast between conditions, thereby enhancing statistical power. On the other hand, prolonging the experiment might have led to fatigue or emotional blunting, particularly in the negative condition. Overall, despite the slight trend in valence ratings, approximately ten trials per condition appear to strike a reasonable balance between experimental control and participant burden.

Physiological responses further support the validity of the paradigm. Participants' heart rate was significantly higher when they spoke about their own experiences compared to when they listened to their partner. While there was no main effect of condition, a significant interaction between condition and speaker role indicated that heart rate was especially elevated during negative conversations in which participants themselves were speaking. Interestingly, the heart rate pattern did not fully mirror the subjective arousal ratings, which also reflected heightened arousal when listening to a partner's negative experience.

This discrepancy may suggest that heart rate is more sensitive to active involvement in the interaction, such as speech production, than to emotional processing alone. These findings are consistent with prior research indicating that speech itself elevates heart rate relative to silence, and that the emotional salience of verbal content further intensifies this effect (Brugnera et al. 2018; Smith et al. 2017). Alternatively, the lack of heart rate acceleration in listeners may reflect the complex nature of physiological emotional contagion. As demonstrated by Dimitroff et al. (2017), observing stress in others does not always result in a mirrored physiological state. Instead, observers may exhibit cardiac deceleration, a response associated with freezing or passive information processing during aversive social observation. The divergence between subjective arousal and heart rate in listeners might therefore indicate that while the emotional salience of the speaker's distress is caught, the physiological manifestation is shaped by the listener's passive role in the dyad. Future research should explicitly test for interpersonal cardiac synchrony to determine if listener physiology tracks the temporal dynamics of the speaker's distress. This approach would allow for a more direct assessment of physiological co‐regulation and contagion.

Heart rate also declined linearly across trials, suggesting physiological habituation to the experimental situation over time. Notably, heart rate was positively correlated with both reported arousal and absolute valence, though both correlations were small in magnitude. Together, these findings underscore the emotional salience of the negative speaker role within the paradigm.

Participants who served as the presenter in a trial were more conversationally dominant, indicating that the study design effectively manipulated speaker and listener roles per trial, allowing for instance research on lead‐follow relationships in social interaction.

Despite the paradigm's strengths, three primary limitations warrant consideration: (1) the current focus on individual‐level validation rather than dyadic synchrony, (2) potential sample bias, and (3) specific design constraints related to topic selection, trial duration, and dyads' baseline familiarity.

Regarding the first limitation, the paradigm's ability to reliably instantiate the psychological and physiological building blocks of interaction is a logically prior step to testing higher‐order phenomena in dual‐person research. However, the current study does not provide empirical evidence of interpersonal synchrony. Instead, we provide a transparent, high‐quality baseline that ensures future observations of synchrony are attributable to the interaction dynamics rather than artifacts of the experimental design.

Regarding the second limitation, the validity of the paradigm is supported by the observation that participants felt more connected to each other when discussing negative experiences and when interacting with someone they felt particularly close to. The effect of discussing a negative topic was especially pronounced when speaking with a close friend. Together, these findings align with the idea that social sharing of emotions is closely linked to social bonding processes (Rimé 2009; Rimé et al. 2020), supporting the validity of the paradigm. Nonetheless, it is important to consider that our sample was likely biased due to the high proportion of female participants and psychology students, two groups that tend to score higher on personality traits like neuroticism, openness to experience, and agreeableness (Vedel 2016). Higher neuroticism is associated with increased emotional reactivity to negative personal experiences and a stronger tendency to ruminate (Muir et al. 2023), which could have amplified the arousal and emotional co‐regulation processes during negative autobiographical recall. Similarly, participants scoring high in openness to experience may have been more willing to engage with the paradigm, while higher agreeableness may have facilitated greater social sensitivity and bonding (Jensen‐Campbell et al. 2010). Thus, our findings may overestimate the extent of these effects compared to a more diverse sample.

Regarding the third limitation, social topics were linked to more negative valence and higher arousal, suggesting that experiences with a social component tend to be particularly emotionally salient. Since about half of the chosen topics were not social in nature, this may explain why the overall negativity of the conversations remained relatively mild. To elicit a stronger negative valence in future studies, participants could be explicitly instructed to recall negative events involving another person.

Overall, 120 s appears to be an appropriate conversation duration for eliciting the desired valence, particularly for neutral topics. However, based on participant feedback and the experimenter's observations, a longer duration would have been beneficial for thoroughly discussing personal events, especially among individuals who are already familiar with each other. Therefore, the current trial length may be particularly suitable for unacquainted individuals but could be extended depending on the sample. Given the statistical analyses commonly used in interpersonal synchrony research, such as wavelet transform coherence (Grinsted et al. 2004), a shorter trial duration should be avoided.

While the use of friend dyads provided a robust environment for validation, we acknowledge that interpersonal familiarity shapes baseline affect and interaction styles. Friends may enter the task with a pre‐existing positive affective tone and established co‐regulation patterns (Tao et al. 2025) that differ from the more cautious, neutral baseline of stranger dyads. While this stability served our validation goals by reducing uncontrolled variance, it limits the direct generalizability of these findings to non‐familiar dyads. Consequently, the emotional intensity observed here may represent an upper bound. Future research is needed to determine if the paradigm retains its efficacy when the buffer of friendship is removed and to disentangle relationship‐specific effects from more general interactional mechanisms.

A key distinction of the present paradigm compared to many existing interaction frameworks is its focus on naturalistic, spontaneous behavior rather than instructed motor coordination. While many paradigms capture nonverbal synchrony by directing participants to perform specific movements, our design allows for the free expression of nonverbal cues (e.g., gestures, facial expressions, and postural shifts) during (semi‐)naturalistic conversation. By recording these sessions via video, the paradigm ensures that these crucial nonverbal channels are preserved. Although the current study focuses on validating the emotional and physiological elicitation of the verbal task, the setup seems suited to provide a rich, multimodal dataset that allows for the future exploration of how nonverbal dynamics emerge organically in response to personal emotional disclosure.

The proposed social interaction paradigm offers several key strengths that enhance its applicability and scientific utility for investigating interpersonal synchrony. First, it is easily understandable and highly practical, making it accessible for both participants and researchers while ensuring smooth implementation in experimental settings. Second, it effectively balances power, standardization, and ecological validity. By incorporating multiple interaction trials, it increases statistical power, while the structured yet naturalistic design maintains experimental control without excessively compromising the authenticity of real‐world social exchanges. Finally, the paradigm is highly flexible, allowing for modifications to experimental conditions, such as replacing negative with positive autobiographical recall, thereby enabling a broader investigation of emotional and interpersonal synchrony across different affective contexts and in diverse populations. We therefore think that this paradigm will serve as a valuable tool for advancing the study of interpersonal synchrony.

## Author Contributions

**Lea Krismann:** data curation, formal analysis, investigation, methodology, writing – review and editing. **Marcel Franz:** conceptualization, methodology, supervision, writing – review and editing. **Fabian Rottstädt:** conceptualization, methodology, writing – review and editing. **Vanessa Nöring:** conceptualization, data curation, formal analysis, investigation, methodology, visualization, writing – review and editing, writing – original draft. **Ilona Croy:** conceptualization, funding acquisition, methodology, supervision, project administration, resources, writing – review and editing.

## Funding

This research is funded by the Federal Ministry of Education and Research (BMBF) projects 01EE2305B and 01EE2505B “DZPG Funding ‐ “Jena location” ‐ CIRC ‐ (Mal)adaptive brain circuits of social interaction, FSU”.

## Disclosure

The authors confirm that the manuscript is an honest, accurate, and transparent account of the study being reported; that no important aspects of the study have been omitted; and that any discrepancies from the study as planned have been explained.

## Ethics Statement

This study was conducted in accordance with the declaration of Helsinki and approved by the Ethics Review Board of the Faculty of Social and Behavioral Sciences of the University of Jena (FSV 22/063). Informed consent was obtained from all individual participants in the study.

## Conflicts of Interest

The authors declare no conflicts of interest.

## Supporting information


**Data S1:** psyp70292‐sup‐0001‐Supinfo.docx.
**Figure S1:** Temporal dynamics of valence across the course of conversation.
**Figure S2:** Valence and arousal across conversation categories.
**Figure S3:** Valence by condition and across mixed‐gender and same‐gender dyads.
**Figure S4:** Arousal by topic presenter and condition across mixed‐gender and same‐gender dyads.
**Figure S5:** Heart rate by condition and topic presenter across mixed‐gender and same‐gender dyads.
**Figure S6:** Dominance ratings by topic presenter and across dyad gender compositions.

## Data Availability

The dataset supporting the findings of this study is publicly available at Open Science Framework (OSF) at https://doi.org/10.17605/OSF.IO/2MXJY. Analysis code and research materials are available from the corresponding author upon request.
