# Supplementary material for: Bridging Lab and Life: A Dual‐Person Paradigm for Social Interaction Research
Source: Psychophysiology. 2026 Apr 8;63(4):e70292. doi: 10.1111/psyp.70292 (PMC13058868; doi:10.1111/psyp.70292)
Supplement: Supplementary file 1 — Data S1: psyp70292‐sup‐0001‐Supinfo.docx. Figure S1: Temporal dynamics of valence across the course of conversation. Figure S2: Valence and arousal across conversation categories. Figure S3: Valence by condition and across mixed‐gender and same‐gender dyads. Figure S4: Arousal by topic presenter and condition across mixed‐gender and same‐gender dyads. Figure S5: Heart rate by condition and topic presenter across mixed‐gender and same‐gender dyads. Figure S6: Dominance ratings by topic presenter and across dyad gender compositions. [file PSYP-63-e70292-s001.docx]

**Supplement**

**Pilot Study**

The primary aim of the pilot study was to design a naturalistic paradigm, which enables to test individuals in different emotional states during a social interaction.

**Methods**: Participants were invited as 14 pairs of strangers (n=28, aged 18-34, mean: 22 ± 4, 14 women) who each performed a ten-minute semi-structured social interaction paradigm in which they shared personal information. The study was approved by the local ethic board of the Friedrich-Schiller University Jena. The interaction was guided by two neutral questions ("*What did you do today?*" [experience of day] and "*What did you eat today?*" [food] ), one emotionally positive question ("*What is your passion?*" [passion]), and one emotionally negative question ("*What was a difficult situation last week?*" [difficult situation]). The participants were instructed to each cover all those conversation categories within the ten-minute interval but could choose the order of the questions. They were also encouraged to ask follow-up questions. These questions were visibly placed in the room during the conversation. For a structured start to the interaction, participants were asked to introduce themselves by sharing their name, age, and profession. The beginning and the end of the 10-min interval was signaled by a beep and a timer was displayed on the screen to indicate the remaining time, using Presentation (Neurobehavioral Systems, 2023). The interaction partners were placed on two chairs at a distance of approximately 90 cm, which were arranged at a diagonal angle to each other. The interaction was video recorded with two cameras, each recording one participant. The experimenter remained present in the room but sat behind a room divider to minimize disturbances.

For coding emotional valence and arousal, we defined conversation sequences in real-time using Mangold INTERACT (Mangold, 2018), marking the time when the dyad began discussing a new question. Each conversation sequence lasted until the initiation of a discussion on a different conversation question. Additionally, the emotional valence of each question was coded as either neutral, negative, or positive. Next, the videos were analyzed to ascertain the conversation question in each sequence and to explore the content regarding the categories passion and difficult situations. For the latter, participants’ responses were transcribed, categorized using a bottom-up approach, and assigned to mutually exclusive categories. Finally, the videos were reanalyzed using the Self-Assessment Manikin (SAM; Bradley & Lang, 1994), subscale valence and arousal, to assess differences in both valence and arousal across the different conversation questions. To evaluate the significance of the mean differences, one-way ANOVAs with planned contrasts were conducted for arousal and valence. Tukey HSD test was analyzed for post hoc comparisons in case of a significant main effect of question.

**Results:** Each dyad covered topics of positive, negative, and neutral valence (Figure 8), but lengths and timing of conversation sequences differed across dyads. The conversation categories differed in valence (F[3, 114] = 91.9, p < 0.001) with medium to large effect size (η² = 0.71). The emotional valence was significantly more negative when instructed to describe a difficult situation as compared to all other conversation categories (p < 0.001). Additionally, emotional valence for passion was significantly higher than for experience of the day (p < 0.001, Figure 9a). The effect of conversation category on arousal was not significant, F(3, 114) = 1.52, p = 0.212, η² = 0.04, Figure 9b). Regarding the specific content of the conversations, participants most frequently mentioned sports when talking about their passion (13 times), followed by their academic field, music, travel, and cooking, each of which was named at least four times. When describing a difficult situation, participants most frequently mentioned university-related challenges (18 times), followed by issues related to social relationships (6 times). All other topics, including travel, loss, moving, discrimination, and work, were mentioned by only one or two participants.

**Discussion:** The paradigm inherently possesses high ecological validity, but it requires adjustments to enhance its standardization. The lack of standardization – especially regarding the stability of temporal windows - presents a significant challenge for data analysis. Other limitations are the rather low difference in arousal, indicating low emotional involvement, which might be partially explained by the nature of the relationship of the interaction partners. Additionally, the lack of self-assessment of arousal and valence limits our findings. We therefore aimed for a higher standardization, more dense self-assessment and dyads who are familiar with each other instead of strangers.

**Dyad Gender Composition**

In this section, we report exploratory analyses extending the models described in the main text. We included gender composition as an additional factor to investigate its influence on the following dependent variables: valence, arousal, heart rate, and dominance.

**Valence:** We conducted a linear mixed model with valence as the dependent variable. Fixed effects included condition (negative vs. neutral; reference: neutral), topic presenter (self vs. other; reference: other), trial number, and gender composition (same-gender vs. mixed-gender; reference: mixed-gender). We included a two-way interaction between condition and topic presenter. Random intercepts were specified for individuals and dyads.

The analysis revealed a low but significant main effect of gender composition (*b* = -0.31, *SE* = 0.13, *t*(67.0) = *-2.38, p* =.02), with participants in same-gender dyads reporting more negative valence than those in mixed-gender dyads. Significant main effects were also observed for condition (negative < neutral; *b* = -2.42, *SE* = 0.07, *t*(2342) = -36.31, *p* < .001) and trial number (*b* = -0.03, *SE* = 0.01, *t*(2342) = -5.96, *p* < .001). The main effect of topic presenter was non-significant (*b* = -0.09, *SE* = 0.07, *t*(2342) = -1.34, *p* = .18) and the condition topic presenter interaction was also non-significant (*b* = -0.16, *SE* = 0.09, *t*(2342) = -1.71, *p* = .09). Fig. 10 displays boxplots and scatterplots of valence by condition and gender composition.

**Arousal:** The arousal model used the same fixed effects and interaction terms as the valence model. Due to a singular fit, the model included a random intercept only for individuals.

Gender composition did not significantly predict arousal (*b* = 0.12, *SE* = 0.19, *t*(136.0) = 0.61, *p* = .54). The effect of trial number was also non-significant (*b* =-0.002, *SE* = 0.005, *t*(2342) = -0.37, *p* = .71). Significant main effects were found for condition (Negative > Neutral; *b* = 1.57, *SE* = 0.08, *t*(2342) = 18.75, *p* < .001), and topic presenter (Self > Other; *b* = 0.42, *SE* = 0.09, *t*(2342) = 4.75, *p* < 0.001). These were qualified by a significant condition and topic presenter interaction (*b* = 0.31, *SE* = 0.12, *t*(2342) = 2.59, *p* < .01). Figure 11 displays boxplots and scatterplots of arousal by topic presenter and condition and across dyad gender composition.

**Heart Rate:** The heart rate model used the same fixed effects and interaction terms as the valence model. Due to a singular fit, the model included a random intercept only for individuals.

The effect of gender composition on heart rate was non-significant, *b* = 4.80, *SE* = 2.43, *t*(102.30) = 1.97, *p* = 0.051. We found significant main effects for topic presenter (Self > Other; *b* = 2.98, *SE* = 0.30, *t*(1313) = 10.03, *p* < 0.001) and trial number (*b* = -0.34, *SE* = 0.02, *t*(1314) = -17.93, *p* < 0.001). The main effect of condition was non-significant (*b* =-0.05, *SE* = 0.27, *t*(1314) = -0.20, *p* = .84). There was a significant condition and topic presenter interaction (*b* = 1.64, *SE* = 0.39, *t*(1312) = 4.18, *p* < 0.001). Figure 12 displays boxplots and scatterplots of heart rate by condition and topic presenter across dyad gender composition.

**Dominance:** We conducted a linear regression with dominance as the dependent variable. Independent variables were topic presenter (self vs. other) and gender composition (same-gender vs. mixed-gender; reference: mixed-gender).

Results: Gender composition had no significant effect on dominance (*b* = -0.03, *SE* = .09, *t*(1239) = -0.315, *p* = .75). Dominance was significantly predicted by topic presenter (*b* = 5.25, *SE* = 0.08, *t*(1239) = 68.09, p < .001, indicating that participants felt more dominant when discussing their own topic. Figure 13 displays boxplots and scatterplots of dominance ratings by topic presenter and across dyad gender composition.


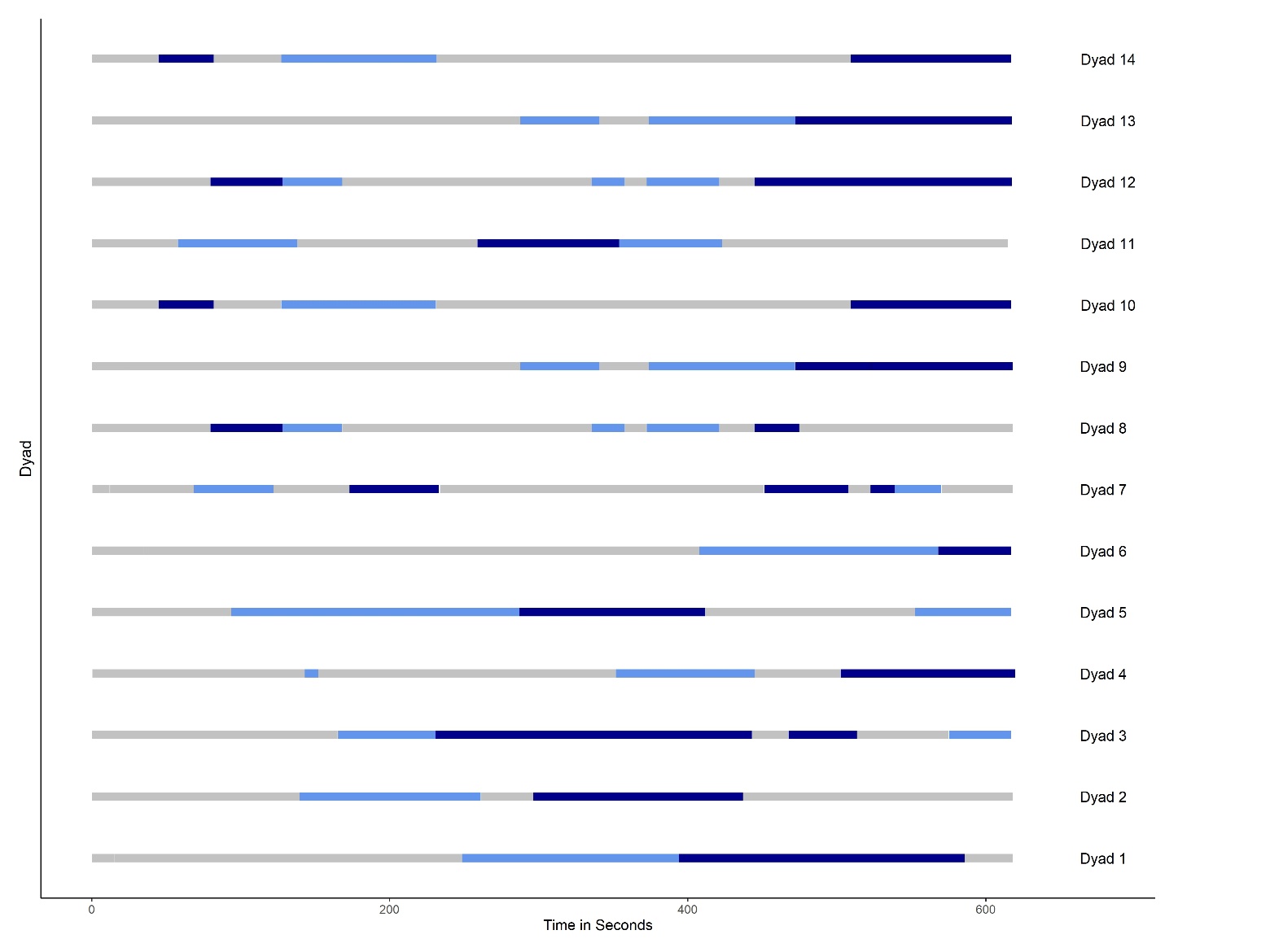


**Figure 8**

Temporal Dynamics of Valence Across the Course of Conversation

*Note.* Temporal dynamic of emotional valence (grey indicating neutral, light blue indicating positive, and dark blue indicating negative valence) during the conversation (time in seconds) for all dyads.


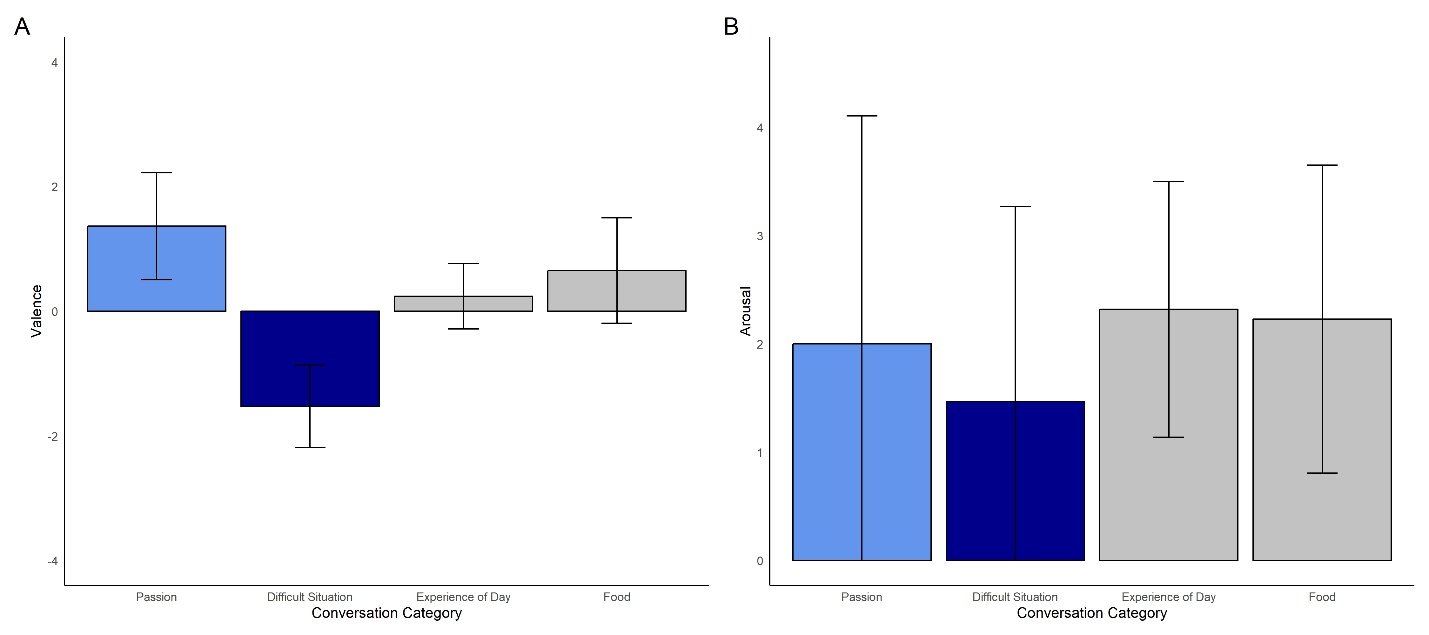


Note. Means and standard deviations for emotional valence (A) and arousal (B) across all conversation categories. To ensure consistency with the main figures, colors represent valence categories: light blue indicates positive valence (passion), dark blue indicates negative valence (difficult situation), and gray indicates neutral valence (experience of day and food). The bars represent the mean values, with error bars indicating the standard deviations. Emotional valence was highest for the conversation category passion (M = 1.36 ± 0.86), followed by food (M = 0.65 ± 0.85) and experience of the day (M = 0.24 ± 0.25) while difficult situation category had the most negative valence (M = -1.53 ± 0.66). Arousal was rather similar between the conditions experience of the day (M = 2.32 ± 1.18), food (M = 2.23 ± 1.42), and passion (M = 2.00 ± 2.11), and difficult situation category eliciting the lowest arousal (M = 1.47 ± 1.80).

**Figure 9**

Valence and Arousal Across Conversation Categories


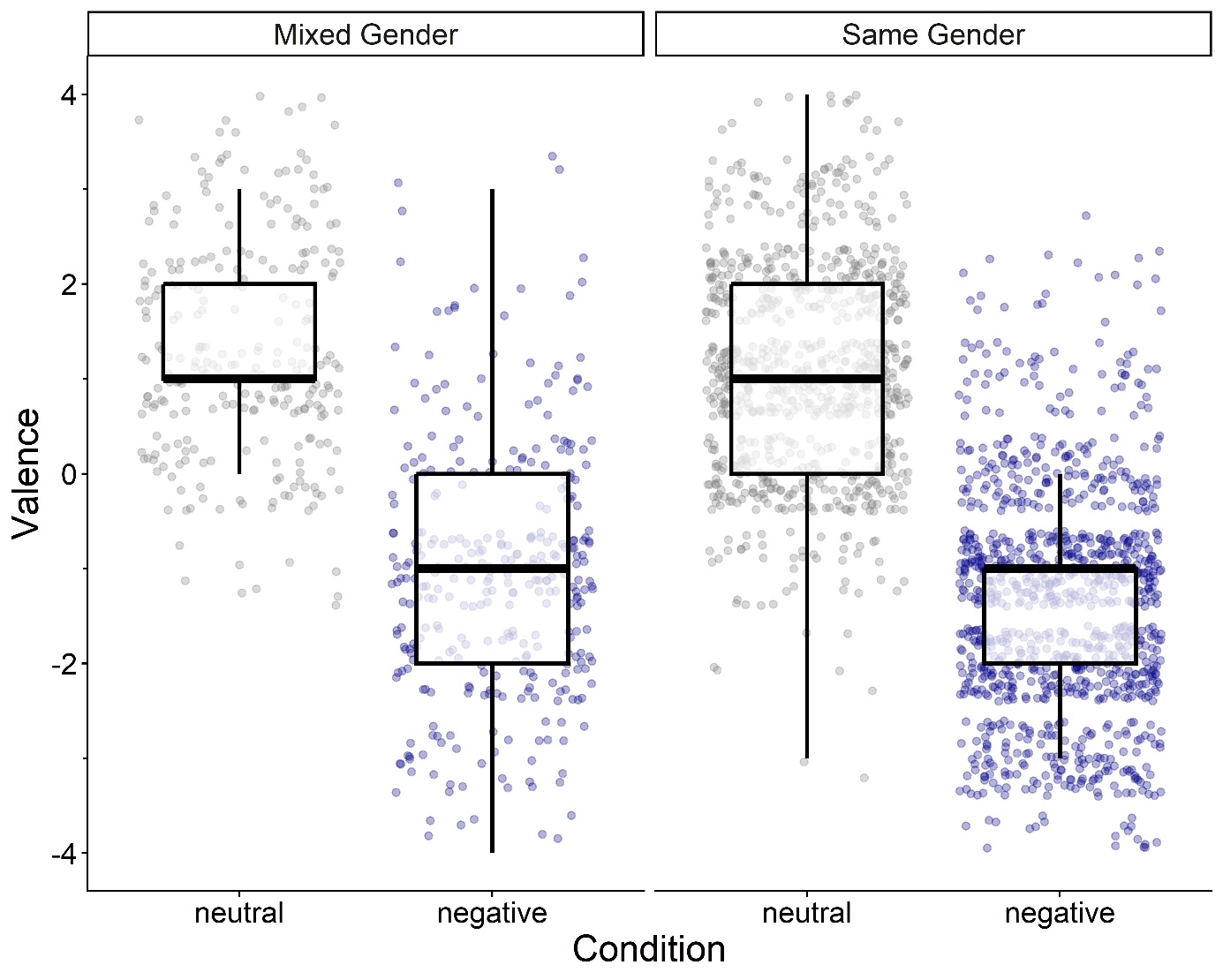


**Figure 10**

Valence by Condition and across Mixed-Gender and Same-Gender Dyads

*Note.* Boxplot and scatterplot of experienced valence by condition and gender composition. Each scatter point represents an individual trial. The boxplot shows the median and interquartile range.


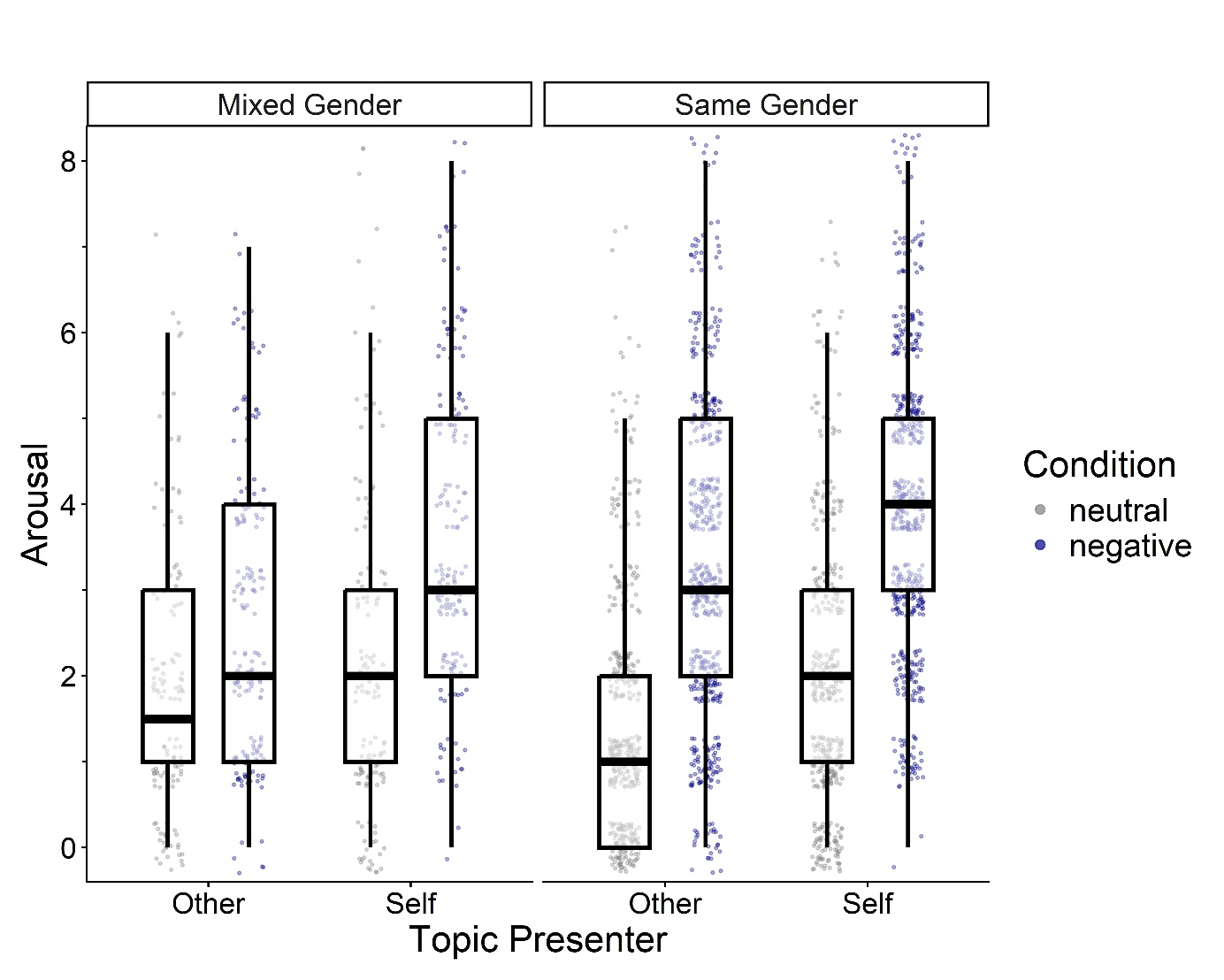
*Note.* Boxplots and scatterplots of arousal by topic presenter and condition and across dyad gender composition. Each scatter point represents an individual trial. The boxplot shows the median and interquartile range.

**Figure 11**

Arousal by Topic Presenter and Condition Across Mixed-Gender and Same-Gender Dyads


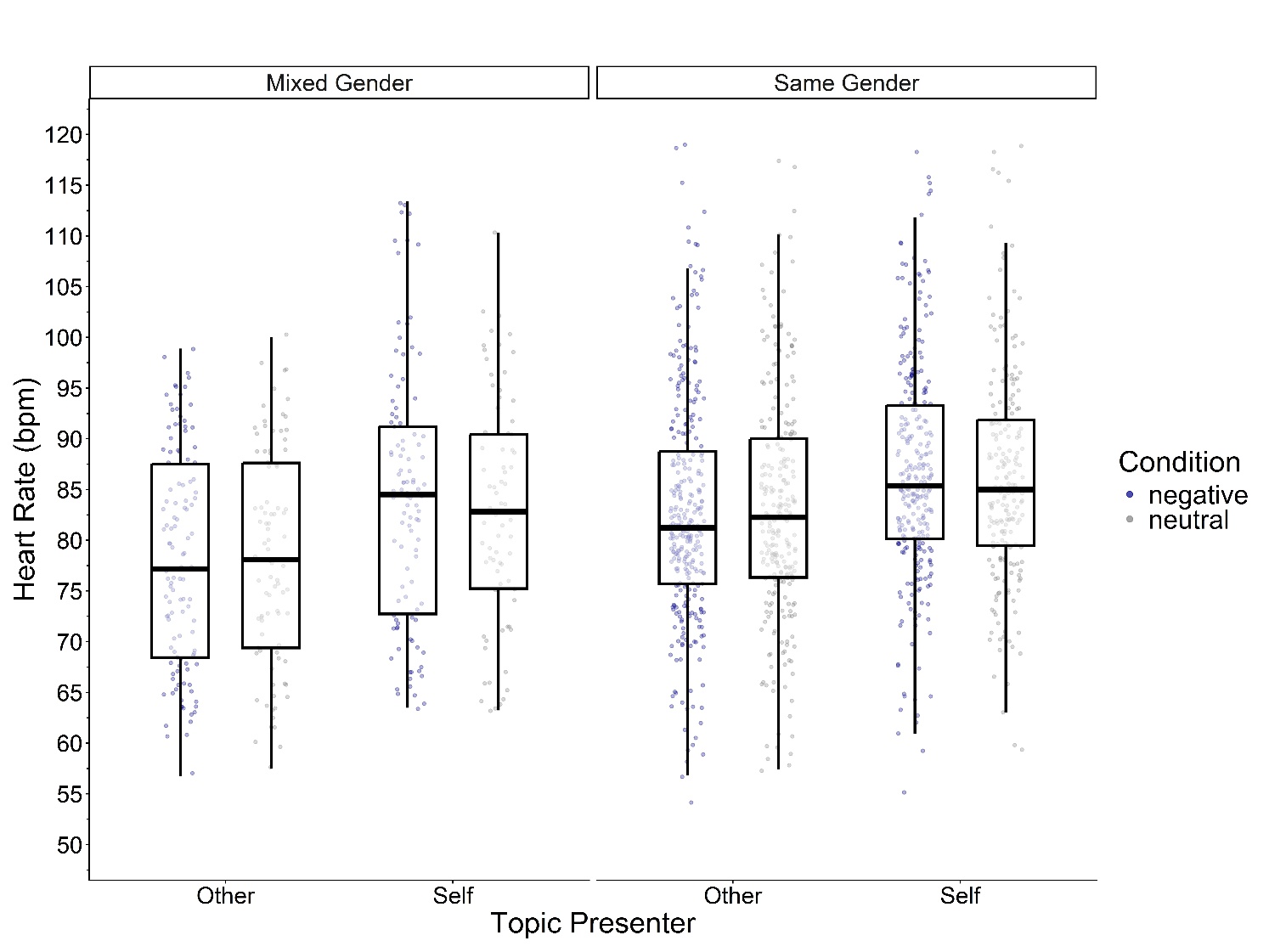
*Note.* Boxplots and scatterplots of heart rate by condition and topic presenter across mixed-gender and same-gender dyads. Each scatter point represents the average heart rate per trial and individual. The boxplot shows the median and quartiles.

**Figure 12**

Heart Rate by Condition and Topic Presenter across Mixed-Gender and Same-Gender Dyads


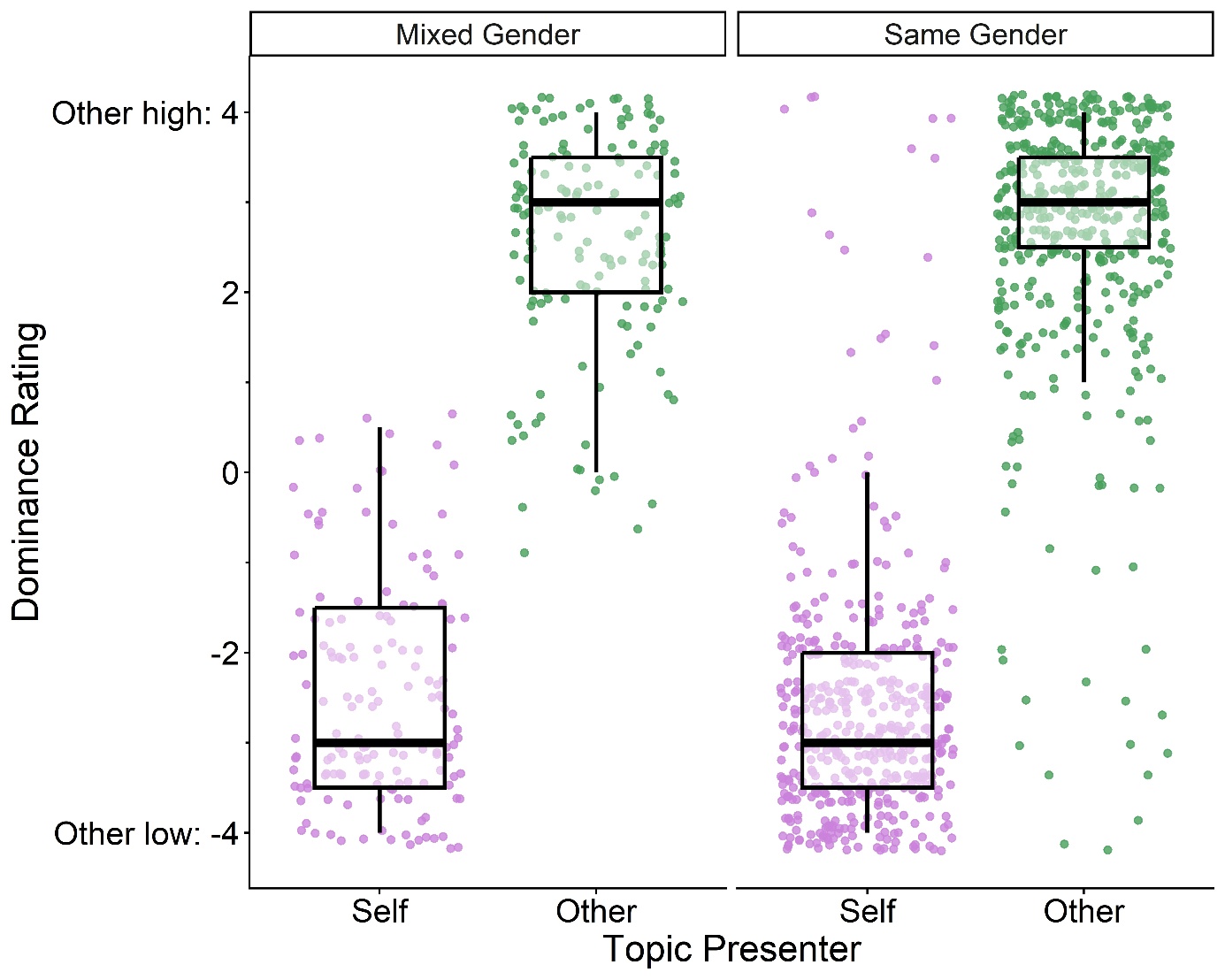


**Figure 13**

Dominance Ratings by Topic Presenter and across Dyad Gender Compositions

*Note.* Boxplots and scatterplots of dominance ratings by topic presenter and across mixed-gender and same-gender dyads. Each scatter point represents a dyad and trial. Negative dominance values indicate low conversational dominance of the other, while positive values indicate high dominance of the other.

**References**

Bradley, M. M., & Lang, P. J. (1994). Measuring emotion: The self-assessment manikin and the semantic differential. *Journal of Behavior Therapy and Experimental Psychiatry*, *25*(1), 49–59. https://doi.org/10.1016/0005-7916(94)90063-9

Mangold, P. (2018). Das Unsichtbare entdecken durch werkzeuggestützte wissenschaftliche Beobachtung. In *Mindful Evolution. Conference Proceedings.* Klinkhardt.

Neurobehavioral Systems. (2023). *Presentation* (Version 23.0) [Computer software].
